# Predictive Biomarkers of Immune Checkpoint Inhibition in Gastroesophageal Cancers

**DOI:** 10.3389/fonc.2020.00763

**Published:** 2020-05-15

**Authors:** Raghav Sundar, Elizabeth C. Smyth, Siyu Peng, Joe P. S. Yeong, Patrick Tan

**Affiliations:** ^1^Department of Haematology-Oncology, National University Health System, Singapore, Singapore; ^2^Cancer and Stem Cell Biology Program, Duke-NUS Medical School, Singapore, Singapore; ^3^Yong Loo Lin School of Medicine, National University of Singapore, Singapore, Singapore; ^4^The N.1 Institute for Health, National University of Singapore, Singapore, Singapore; ^5^Department of Oncology, Cambridge University Hospitals NHS Foundation Trust, Cambridge, United Kingdom; ^6^University Medicine Cluster, National University Health System, Singapore, Singapore; ^7^Division of Pathology, Singapore General Hospital, Singapore, Singapore; ^8^Institute of Molecular Cell Biology, Agency of Science, Technology and Research (ASTAR), Singapore, Singapore; ^9^Cancer Science Institute of Singapore, National University of Singapore, Singapore, Singapore; ^10^Genome Institute of Singapore, Agency for Science, Technology and Research, Singapore, Singapore; ^11^SingHealth/Duke-NUS Institute of Precision Medicine, National Heart Centre Singapore, Singapore, Singapore

**Keywords:** immunotherapy, biomarker, gastric cancer, esophageal cancer, gastroesophageal cancer, immune checkpoint inhibition, predictive biomarker, precision oncology

## Abstract

Immune checkpoint inhibition has transformed cancer treatment. For gastroesophageal cancer, this class of drugs have demonstrated durable responses and survival benefit in a subgroup of patients, resulting in regulatory approval. However, several recent randomized phase III studies in gastroesophageal cancer have reported negative results, blunting initial enthusiasm. Identification and validation of predictive biomarkers with appropriate patient selection for benefit from immunotherapy is an area of intense research with novel concepts rapidly emerging. In this review we describe the latest immune checkpoint inhibitor trials which have been reported in gastroesophageal cancers with a focus on predictive biomarkers. We also explore novel biomarkers being developed to improve precision oncology for immunotherapy in gastroesophageal cancers.

## Introduction

Gastroesophageal cancers (GEC) are a leading cause of cancer morbidity and mortality globally. GEC may be classified as gastric cancer (GC) and esophageal cancer (EC) based on anatomical location. GC is the third most common cause of cancer-related death, while EC is sixth (1). Approximately 1.3 million patients die from GEC annually (GLOBOCAN database). Esophageal cancers are further subtyped by histology into esophageal adenocarcinoma (EAC) and esophageal squamous cell cancer (ESCC) (2). Management of GEC has evolved over time, with early clinical trials of chemotherapy and radiation therapy combining both GC and EC (EAC and ESCC) in the same trials (3). However, contemporary trials with newer agents are designed with more specific inclusion criteria for GC or ESCC only (4).

GC is characterized by inter and intra-tumoral heterogeneity, leading to significant hurdles in the advance of precision oncology in this tumor type (5). Chemotherapy remains the standard of care (SOC) for metastatic GC. Although treatment regimens differ between regions and institutes, platinum and 5-fluoropyrimidine (5FU) combination regimens are generally preferred in first-line treatment (6). Median overall survival in contemporary clinical trials for platinum/5FU is 11 months (7). Multiple targeted therapy phase III trials have failed for drugs targeting EGFR, VEGF and PARP (8–11). Trastuzumab was one of the first targeted therapies that received Food and Drug Administration (FDA) approval in combination with cytotoxic chemotherapy for a biomarker selected population (HER2 positive) in GC (12). HER2 remains one of the few biomarkers of clinical value for the treatment of metastatic GC. The Cancer Genome Atlas (TCGA) classifies GC into four main subgroups—chromosomally instable (CIN), genomically stable (GS), high microsatellite instability (MSI), and Epstein-Barr virus (EBV) positive tumors (13). However, until the recent emergence of immunotherapy, this classification has not been incorporated into clinical practice.

Precision oncology in metastatic EC has also made slow progress, with few in-roads for targeted therapy agents. The TCGA classification of EC describe genetic and epigenetic differences between EAC and ESCC (2). Epigenetically, EAC resembles the CIN subtype of GC. The incidence of EBV and MSI is extremely low in EC (<1%) (14). There are no targeted therapies approved for the treatment of metastatic EC. Before the immunotherapy era, all patients with metastatic EC were treated with chemotherapy, with no predictive biomarkers to guide treatment. However, contemporary trials in EC have integrated biomarker discovery and validation, particularly those involving immunotherapy (15).

Pathways that regulate the immune system are called immune checkpoints. These pathways have evolved due to the biological necessity of self-tolerance. However, several cancers coopt immune checkpoints as a mechanism of immune-editing and immune-evasion (16). The dependence of cancer on these pathways can be therapeutically exploited, by targeting specific checkpoints [immune checkpoint inhibitors (ICI)]. CTLA-4 is a receptor expressed on regulatory T-cells (Tregs). It functions as a negative co-stimulator, when bound to CD80 or CD86 on antigen presenting cells, leading to modulation of immune response (17). Ipilimumab is an anti-CTLA4 monoclonal antibody, and is the first ICI to gain regulatory approval in cancer therapy (for the treatment of melanoma) (18). Programmed cell death 1 (PD-1) is another negative co-stimulatory transmembrane protein expressed on T-cells, B-cells, and NK cells. PD-1 binds to PD-1 ligand (PD-L1) and PD-L2. PD-L1 is expressed on tumor cells and multiple tissue types (19). The interaction between PD-1 and PD-L1/2 promotes peripheral T effector cell modulation, inhibits tumor cell apoptosis, and increases conversion of T effector cells to Treg cells (17). Blockade of the PD-1 axis with anti-PD-1 or anti-PD-L1 monoclonal antibodies restores anti-tumor immune responses and leads to tumor regression. Several anti-PD-1 and anti-PD-L1 antibodies are now approved as single-agents and in combination with other drugs for the treatment of multiple tumor types including lung cancer, melanoma, renal cell carcinoma, hepatocellular carcinoma, and others (20, 21). In GEC, the anti-PD-1 antibodies pembrolizumab and nivolumab have demonstrated survival benefit and gained regulatory approval. However, recently, several phase III trials across multiple tumor types (including GEC) have reported negative outcomes, with ICI failing to demonstrate superiority over standard-of-care (SOC) therapies (22–28). These studies highlight the importance of biomarker development and appropriate patient selection for ICI.

In this review we first summarize the major immunotherapy trials in GEC, including randomized phase III trials and major phase II trials (leading to registration) reported before January 2020. Next, we focus on biomarkers that have been studied within these trials, and finally we highlight emerging novel predictive biomarkers of immunotherapy benefit in GEC.

## PD-L1 Immunohistochemistry

**Figure 1:**
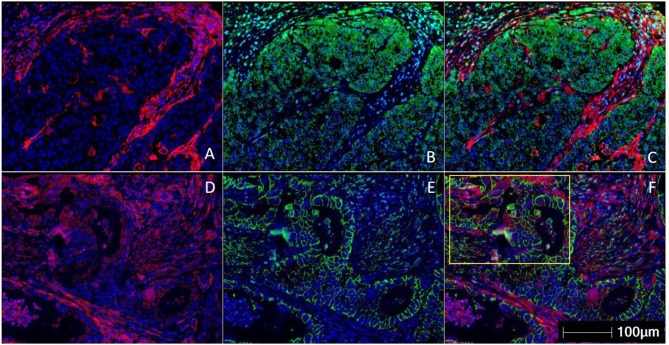
Multiplex immunohistochemistry/ immunofluorescence (mIHC/IF) staining of gastric carcinoma. Two gastric cancer samples are labeled with PD-L1 (Clone 22C3) (red), EpCAM (green), and DAPI (blue) by using mIHC/IF. EpCAM is a cell surface protein commonly expressed in gastric cancer cells. DAPI is a nuclear counterstain. **(A–C)** PD-L1 staining (in red) can be observed only on the immune cells surrounding the tumor nests. There is minimal PD-L1 expression within the tumor cells. Image A is mIHC/IF imaging of PD-L1 + DAPI, Image B is EpCAM + DAPI and Image C is PD-L1 + EpCAM + DAPI. **(D–F)** PD-L1 staining (in red) can be seen in both tumor compartment (majority of the positive cells are located in the yellow highlighted box) and surrounding immune cells. Image D is mIHC/IF imaging of PD-L1 + DAPI, Image E is EpCAM + DAPI and Image F is PD-L1 + EpCAM + DAPI. These images highlight the importance of considering immune-cell expression along with tumor expression of PD-L1 IHC, and the differences between TPS and CPS scores in gastric cancer. Representative images are shown with HALO^TM^ pathology analysis software. (Magnification: 200X).

The measurement of intra-tumoral PD-L1 expression using immunohistochemistry (IHC) is one of the earliest biomarkers developed for predicting benefit from ICI (29). A method of scoring PD-L1 called the tumor proportion score (TPS) was developed for lung cancer, involved measurement of PD-L1 expression only within the tumor cells (30). The TPS score was presented as the ratio of the number of PD-L1–expressing tumor cells to all tumor cells present. The TPS method was found to be inadequate in GEC, and it was found that measuring the expression of PD-L1 in the immune cells surrounding the tumor was especially important in GEC (31). The combined positive score (CPS) was developed to consider the expression of PD-L1 on tumor cells and immune cells combined. CPS is the ratio of the number of all PD-L1–expressing cells (tumor cells, lymphocytes, macrophages) to the number of all tumor cells (32) (Figure 1). The PD-L1 CPS score was developed using the 22C3 assay as a companion diagnostic for pembrolizumab in GC and has been FDA approved (33).

## Key Trials of Immune Checkpoint Inhibition in Gastroesophageal Cancers

Early data for ICI in GEC came from large basket studies of pembrolizumab that included various advanced, solid tumors expressing PD-L1. In the phase 1b KEYNOTE-012, 39 patients with metastatic GC were included, and achieved an objective response rate (ORR) of 22%, with a median duration of response of 40 weeks (31). The phase 1b KEYNOTE-028 included 28 patients with metastatic EC. Both EAC and ESCC were included and an ORR of 30% was achieved, with a median duration of response of 15 months (34). These encouraging early results led to larger, tumor-type specific single-arm phase II and randomized phase III trials.

### Metastatic Third-Line Gastric Cancer

#### ATTRACTION-2 (Nivolumab)

ATTRACTION-2 was a randomized phase III study run in Japan, South Korea, and Taiwan (35). Patients with metastatic GC that had progressed on at least 2 prior lines of therapy were eligible for the study. In total 493 patients were randomized to intravenous (i.v.) nivolumab 3 mg/kg every 2 weeks vs. placebo. Nivolumab demonstrated an overall survival (OS) benefit of 5.3 months compared to 4.1 months for placebo (Table 1). More importantly, there was a group of patients with refractory disease that appeared to have durable benefit from treatment, with 1-year and 2-year OS being superior in the nivolumab arm [27 vs. 12% (1-year OS); 11 vs. 3% (2-year OS)] (37). This trial led to the approval of nivolumab in the third-line metastatic GC in Japan and Korea. The study was conducted in a purely Asian population, raising the concern of applicability in other populations. GC in Asian patients have been shown to have a significantly different immune signature compared to GC in non-Asian populations, with enrichment in Tregs (38). Fortunately, results from trials including predominantly non-Asian populations addressed these questions.

**Table 1:** Key 3rd line trials for metastatic gastric cancer.

**Name of trial**	**ICI**	**Comp**	**N**	**OS (months)**			**PFS (months)**			**ORR (%)**		**References**
				**ICI**	**Comp**	**HR**	**ICI**	**Comp**	**HR**	**ICI**	**Comp**	
ATTRACTION-2	Nivo	Placebo	493	5.3	4.1	0.6	1.6	1.4	0.6	11	0	(35)
KEYNOTE-059	Pembro	NA	259	5.6	NA	NA	2.0	NA	NA	12	NA	(36)
JAVELIN 300	Avel	Pacli or Irino	371	4.6	5.0	1.1	1.4	2.7	1.7	2	4	(23)

*ICI, immune checkpoint inhibitor; Comp, comparator arm; N, number of patients enrolled in the study; ORR, objective response rate; nivo, nivolumab; pembro, pembrolizumab; pacli, paclitaxel; avel, avelumab; irino, irinotecan*.

#### KEYNOTE-059—Cohort 1 (Pembrolizumab)

KEYNOTE-059 was a multi-cohort study of patients with metastatic GC treated with pembrolizumab. Cohort 1 included patients with metastatic GC that had progressed on at least 2 prior lines of therapy, and were treated with i.v. pembrolizumab 200 mg (flat dose) every 3 weeks. This was a single-arm cohort with all patients treated with pembrolizumab, conducted in 16 countries across the globe (36). Of the 259 patients enrolled, objective response rate (ORR) was 12% and median OS was 5.6 months (Table 1). The results from this study led to the FDA approval of pembrolizumab for third-line metastatic GC. Patients in the KEYNOTE-059 Cohort 1 were predominantly non-Asian (>80%). With similar ORR, median PFS and OS in KEYNOTE-059 and ATTRACTION-2 (Table 1), concerns in the differential responses to immunotherapy between Asian and non-Asian GC populations were mitigated.

#### JAVELIN 300 Gastric Cancer (Avelumab)

JAVELIN 300 was a randomized phase III trial run globally, for metastatic GC patients who had failed at least 2 prior lines of systemic therapy (23). Patients were randomized to i.v. avelumab (an anti-PD-L1 monoclonal antibody) 10 mg/kg every 2 weeks or physicians' choice of chemotherapy (paclitaxel or irinotecan). The study randomized 371 patients, and failed to achieve its primary endpoint of OS. There was no difference in OS between avelumab or chemotherapy (4.6 vs. 5.0 months) (Table 1). PFS was better in the chemotherapy arm, compared to avelumab [2.7 vs. 1.4 months, hazards ratio (HR) = 1.73].

The results from these third-line studies have led to the incorporation of pembrolizumab and nivolumab into routine clinical practice for GC. It is interesting to note the control chemotherapy arm in the “negative” JAVELIN study had a numerically higher PFS, and similar OS compared to nivolumab in the “positive” ATTRACTION-2 study. This highlights the importance of an appropriate control arm in designing clinical trials.

### Metastatic Third-Line Esophageal Cancer

#### KEYNOTE-180 (Pembrolizumab)

KEYNOTE-180 was a single-arm study of metastatic EC, that had been treated with at least 2 prior lines of systemic therapy (39). In total 121 patients were treated with pembrolizumab (63 with ESCC and 58 with EAC). ORR was 14% for ESCC and 5% for EAC. Median PFS was 2.0 months and median OS was 5.8 months.

### Metastatic Second-Line Gastric Cancer

#### KEYNOTE-061 (Pembrolizumab)

KEYNOTE-61 was a randomized phase III study of pembrolizumab against weekly paclitaxel for patients that had progressed on 1 prior line of chemotherapy containing a platinum and 5-fluouracil (5-FU) doublet (25). The study randomized 592 patients in total, with the first 489 patients (83%) being enrolled regardless of PD-L1 status. An interim review by an independent data-monitoring committee recommended that the enrollment for the remaining patients were restricted to tumors with a PD-L1 combined positive score (CPS) of 1 or more, based on poorer outcomes of patients with a CPS of 0 with pembrolizumab. The study failed to demonstrate a statistically significant survival difference between pembrolizumab and paclitaxel chemotherapy. Median OS was 9.1 months for pembrolizumab vs. 8.3 months for paclitaxel. Progression free survival (PFS) was 1.5 vs. 4.1 months, in favor of paclitaxel. The study however, had an interesting phenomenon of the survival curves crossing between the two arms of the trial (for OS and PFS at ~8 to 10 months). The crossing of the survival curves violates the assumption of proportional hazards and does not yield a meaningful analysis using Cox regression (40). A small proportion of patients have a prolonged and durable benefit from pembrolizumab, with the survival curve reaching a plateau at 20 months (not observed in the chemotherapy arm). However, a larger proportion of patients received third-line treatment in the paclitaxel arm compared to pembrolizumab (58 vs. 46%). This highlights the importance of disease control with chemotherapy preventing rapid deterioration of function and performance status precluding further lines of treatment. Identification of a negative predictive biomarker of immunotherapy could potentially help these patients avoid ineffective therapy.

### Metastatic Second-Line Esophageal Cancer

#### KEYNOTE-181 (Pembrolizumab)

The KEYNOTE-181 study was restricted to EC patients only, included both EAC and ESCC as well as Siewart's type I gastroesophageal junction (GEJ) tumors (15). Patients that had progressed on one prior line of the chemotherapy were randomized to pembrolizumab or physician's choice of chemotherapy (paclitaxel, docetaxel or irinotecan). The study was designed with three co-primary endpoints: OS in a) patients with a PD-L1 CPS ≥ 10; b) patients with ESCC; c) patients in the intention to treat (ITT), entire trial population. The study randomized 628 patients and met only one of the three primary endpoints (PD-L1 CPS ≥ 10 subgroup). In the ITT population, there was no difference in survival between pembrolizumab and chemotherapy (median OS 7.1 months in both arms) (Table 2). Similarly, there was no statistically significant difference in survival in the ESCC subpopulation (8.2 vs. 7.1 months). In the CPS ≥ 10 subgroup, pembrolizumab had a significant improvement in OS over paclitaxel (9.3 vs. 6.7 months). On the basis of these results, pembrolizumab was granted FDA approval for EC with CPS ≥ 10.

**Table 2:** Key 2rd line trials for metastatic esophageal cancer.

**Name of trial**	**ICI**	**Comp**	**N**	**OS (months)**			**PFS (months)**			**ORR (%)**		**References**
				**ICI**	**Comp**	**HR**	**ICI**	**Comp**	**HR**	**ICI**	**Comp**	
ATTRACTION-3	Nivo	Pacli or Doce	419	10.9	8.4	0.77	1.7	3.4	1.1	19	22	(41)
KEYNOTE-181	Pembro	Pacli or Doce or Irino	628	7.1	7.1	0.89	2.1	3.4	1.1	13	7	(15)

*ICI, immune checkpoint inhibitor; Comp, comparator; N, number of patients enrolled in the study; ORR, objective response rate; nivo, nivolumab; pembro, pembrolizumab; pacli, paclitaxel; doce, docetaxel; irino, irinotecan*.

#### ATTRACTION-3 (Nivolumab)

This study was specific only for ESCC, and randomized 419 patients to nivolumab or physicians choice of paclitaxel or docetaxel in the second-line (41). The study achieved its primary endpoint, demonstrating a statistically significant improvement in OS for nivolumab over chemotherapy (10.9 vs. 8.4 months). The improvement in OS was found despite no difference in PFS or ORR between nivolumab and chemotherapy (Table 2). Prespecified analysis of health-related quality of life was better for nivolumab compared to chemotherapy, which is of importance given a proportion of patients continue with nivolumab for a prolonged period of time (42).

The results of these second line studies have resulted in the incorporation of pembrolizumab and nivolumab into routine clinical practice for EC.

### Metastatic Gastric Cancer First-Line

#### KEYNOTE-059—Cohort 2 and 3 (Pembrolizumab)

KEYNOTE-059 was a multi-cohort study for metastatic GC, treated with pembrolizumab. Cohort 2 was a single-arm cohort for first-line metastatic GC, treating patients with i.v. pembrolizumab 200 mg every 3 weeks, in combination with cisplatin and infusional 5-FU or capecitabine (43). This cohort included 25 patients, ORR was 60%, median PFS was 6.6 months, and median OS was 13.8 months. Cohort 3 was a single-arm cohort including patients with untreated metastatic GC, and a PD-L1 CPS ≥ 1. Patients were treated with i.v. pembrolizumab 200 mg every 3 weeks. In this cohort (*n* = 31) of single agent pembrolizumab, ORR was 26%, median PFS was 3.3 months and median OS was 20.7 months.

#### KEYNOTE-062 (Pembrolizumab)

KEYNOTE-062 was a three arm randomized phase III study with multiple co-primary end- points conducted globally for patients with untreated metastatic GC (28). In total, 763 patients were randomized into 3 arms: pembrolizumab single-agent, chemotherapy (5-FU + platinum doublet) alone, and pembrolizumab + chemotherapy. Based on the KEYNOTE-061 data, this study was designed to only include PD-L1 CPS ≥ 1 population. Key co-primary end-points included non-inferiority of pembrolizumab single-agent to chemotherapy for OS, and superiority of pembrolizumab + chemotherapy over chemotherapy alone for OS. Similar to other KEYNOTE studies, multiple co-primary endpoint testing required α splitting and stringent *p*-values to achieve the primary endpoints. The study achieved one of its co-primary endpoints: pembrolizumab was found to be non-inferior to chemotherapy (median OS 10.6 vs. 11.1 months, HR 0.91). Like KEYNOTE-061, there was a “crossing of the curves” seen at 12 months. One-year OS for pembrolizumab vs. chemotherapy was 47 vs. 46%, while 2-year OS was 27 vs. 19%. The study failed to meet several other primary endpoints. PFS of pembrolizumab was inferior to chemotherapy (2.0 vs. 6.4 months, HR 1.66). ORR was also inferior for pembrolizumab (15 vs. 37%). Pembrolizumab + chemotherapy was not superior to chemotherapy alone (median OS 12.5 vs. 11.1 months, HR 0.85). Interestingly, the 2-year OS of the pembrolizumab + chemotherapy arm was numerically lower than the single agent-pembrolizumab arm (24 vs. 27%).

#### JAVELIN Gastric 100 (Avelumab)

The JAVELIN Gastric 100 study was a randomized phase III trial of avelumab administered as switch maintenance therapy in patients with metastatic GC following first-line chemotherapy (44). In total, 805 patients were randomized after 12 weeks of induction chemotherapy (FOLFOX or XELOX) to either avelumab 10 mg/kg every 2 weeks or continuation of first-line chemotherapy. Patients must have had an ORR of stable disease or better with first-line chemotherapy prior to randomization. The study failed to meet its primary endpoint of OS benefit for avelumab. The median OS was similar between avelumab and chemotherapy (10.4 vs. 10.8 months, HR 0.91). PFS was also similar between avelumab and chemotherapy (3.2 vs. 4.4 months, HR 1.04), as was ORR (13 vs. 14%).

The role of ICI in first-line for GC remains controversial based on these trial results. Despite KEYNOTE-062 achieving one of its primary endpoints, to date, there is no regulatory approval for the use of ICI in the first-line. Results from other first-line studies such as ATTRACTION-4 and CheckMate-649 are awaited (45, 46).

### Ipilimumab in Gastric Cancer

The role of ipilimumab in switch maintenance after first-line chemotherapy was tested in a randomized phase II study (47). Patients with metastatic GC that had achieved at least stable disease with first-line chemotherapy were randomized to ipilimumab or continuation of chemotherapy. In total 114 patients were randomized, and the study failed to meet its primary endpoint of PFS. The median PFS of ipilimumab was inferior to chemotherapy (2.9 vs. 4.9 months, HR 1.44), while OS was similar (12.7 vs. 12.1 months).

CheckMate-032 was a study that included metastatic GC that had progressed on at least one line of treatment (48). Patients were randomized to one of three arms: i.v. nivolumab 3 mg/kg every 2 weeks (NIVO3), nivolumab 1 mg/kg + ipilimumab 3 mg/kg every 3 weeks for 4 cycles (NIVO1 + IPI3) or nivolumab 3 mg/kg + ipilimumab 1 mg/kg every 3 weeks for 4 cycles (NIVO3 + IPI1). NIVO1 + IPI3 and NIVO3 + IPI1 regimens continued with nivolumab 3 mg/kg every 2 weeks after the 4 cycles of combination therapy. In total 160 patients were treated in the study. ORR was 12% (NIVO3), 24% (NIVO1 + IPI3), and 8% (NIVO3 + IPI1), respectively. Median PFS was 1.4 months (NIVO3), 1.4 months (NIVO1 + IPI3), and 1.6 months (NIVO3 + IPI1). The median OS was 6.2 months (NIVO3), 6.9 months (NIVO1 + IPI3), and 4.8 months (NIVO3 + IPI1). There is no regulatory approval for the use of ipilimumab in GEC to date.

## Biomarkers of Immune Checkpoint Inhibition in Metastatic Gastroesophageal Cancer

### PD-L1 Immunohistochemistry

Early development of PD-L1 IHC was conducted in lung cancer. Trials of various anti-PD-1/anti-PD-L1 antibodies incorporated different methods and techniques of PD-L1 IHC measurement for correlative biomarker development (49). Based on these trial results, different monoclonal antibodies and platforms such as the PD-L1 22C3 pharmDx assay (22C3), 28-8 pharmDx assay (28-8), SP263 assay (SP263) and SP142 assay (SP142) have been approved as companion/complementary diagnostics for nivolumab, pembrolizumab and atezolizumab for lung cancer (30). The predictive value of PD-L1 IHC to ICI has been variable across trials. In GC, CPS ≥ 1 occurs in more than half the patients, while TPS ≥ 1% occurs only in 12.5% of patients (32). Initial trials of GC did not select for patients based on PD-L1 status, but retrospective *post-hoc* analyses were performed in these trials to correlate CPS score with response rates and survival. In lung cancer, trials have studied the efficacy of ICI at different TPS dichotomies (e.g. ≥50 vs. <50% and ≥1 vs. <1%) (50). In GEC, CPS ≥ 1 and CPS ≥ 10 scores have been explored as important cut-offs to subclassify patients and these levels have been studied in greatest depth in clinical trials using pembrolizumab.

#### Analysis of Major Pembrolizumab GEC Trials Based on CPS Score

In the KEYNOTE-059 study, of the 259 patients included, 57% had a PD-L1 CPS ≥ 1 (51). Patients that were CPS ≥ 1 had a significantly higher ORR compared to CPS 0 (16 vs. 6%). However, in both CPS 0 and CPS ≥ 1 subgroups, 3 complete responses (CR) were detected, and median OS was similar between both groups (5.8 vs. 4.9 months) (Table 3). In the KEYNOTE-061 study, the original trial design did not preselect patients based on CPS score (25). After 489 patients (out of 983 in total screened) were enrolled, the independent data monitoring committee recommended that only patients with CPS ≥ 1 were included in the study. The co-primary end points were specified to analyze OS and PFS in the CPS ≥ 1 population of the trial. Of the 592 patients randomized in the study, 395 were CPS ≥ 1. Pembrolizumab did not improve OS in the CPS ≥ 1 population (9.1 vs. 8.3 months, HR 0.82). In *post-hoc* unplanned analysis, patients with CPS ≥ 10 had an improved OS with pembrolizumab compared to paclitaxel (10.4 vs. 8 months) (Table 3). Although not reported with statistical analyses, inspection of the survival curves of the CPS <1 population in KEYNOTE-061 suggests detriment for patients treated with pembrolizumab compared to paclitaxel.

**Table 3:** Results of major pembrolizumab trials based on CPS score.

**Name of trial**	**Line of Rx**	**CPS prevalence**		**OS (months)**			**PFS (months)**			**ORR (%)**			**References**
		**≥1**	**≥10**	**0**	**≥1**	**≥10**	**0**	**≥1**	**≥10**	**0**	**≥1**	**≥10**	
KEYNOTE-059	≥3	57%	NA	4.9	5.8	NA	NA	NA	NA	6	16	NA	(36)
KEYNOTE-061	2	66%	18%	4.8	9.1	10.4	NA	1.5	NA	2	16	9	(25)
KEYNOTE-062 (Pembro)	1	All	36%^*^	NA	10.6	17.4	NA	2.0	2.9	NA	15	25	(28)
KEYNOTE-062 (Pembro + chemo)	1	All	36%^*^	NA	12.5	12.3	NA	6.9	5.7	NA	49	53	(28)
KEYNOTE-181 (EC)	2	NR	35%	7.1^+^		9.3	2.1^+^		2.6	13^+^		22	(15)
KEYNOTE-180 (EC)	≥3	NR	48%	NR		6.3	NR		2.0	NR		14	(39)

* *this is the prevalence of CPS ≥ 10 within this trial, which is a biomarker selected population of CPS ≥ 1*.

+ *this trial did not differentiate between CPS 0, and CPS ≥ 1, and the survival/response rates reported here is for the entire trial population (regardless of CPS score)*.

*Pembro, pembrolizumab; chemo, chemotherapy; EC, esophageal carcinoma; NA, not applicable; NR, not reported*.

In the KEYNOTE-062 study, based on results from earlier KEYNOTE studies, patients were restricted only to the CPS ≥ 1 population (28). In unplanned, *post-hoc* analysis, pembrolizumab had significantly improved survival compared to chemotherapy in the CPS ≥ 10 subgroup (17.4 vs. 10.8 months). In the pembrolizumab + chemotherapy arm, similar benefit was not demonstrated. In the CPS ≥ 10 subgroup, there was no improvement in survival for pembrolizumab and chemotherapy compared to chemotherapy alone (12.3 vs. 10.8 months) (Table 3).

In the EC study KEYNOTE 181, patients were not preselected for esophageal tumor subtype or CPS score. Based on the results of the KEYNOTE-061 study, the trial had a pre-specified co-primary endpoint to study the CPS ≥ 10 population independently (15). In this study, 35% of tumors were CPS ≥ 10 and 64% were ESCC. Survival was significantly higher in the CPS ≥ 10 population for pembrolizumab vs. chemotherapy (9.3 vs. 6.7 months, HR 0.69) (Table 3).

#### PD-L1 as a Biomarker in Nivolumab and Avelumab Trials

In the ATTRACTION-2 study of nivolumab in third-line metastatic GC patients, PD-L1 IHC was measured using the 28-8 assay, and defined as positive if ≥1% of tumor cells demonstrated staining (approximately equivalent to TPS ≥ 1%) (35). PD-L1 results were not mandated in the inclusion and only 192 of 493 (39%) had IHC results available. Based on this assay, and definition of PD-L1 positive, only 26 (14%) of patients were defined as PD-L1 positive. In PD-L1 positive tumors median OS was 5.2 and 3.8 months for nivolumab and placebo (HR 0.5), while in the PD-L1 negative tumors median OS was 6.1 and 4.2 months for nivolumab and placebo, respectively (HR 0.72). Given the current understanding of the need to measure immune cell expression of PD-L1 to have a more reliable measure of PD-L1 expression in GC (i.e., measuring CPS instead of TPS), these results must be interpreted with caution and are unlikely to be clinically relevant. In ATTRACTION-3, similar to ATTRACTION-2, PD-L1 IHC was measured using the 28-8 assay, in tumor cells only (41). ATTRACTION-3 was restricted only to patients with ESCC, and approximately half the patients had a PD-L1 expression ≥ 1 and 30% of tumors had PD-L1 expression ≥ 10%. Median OS in the PD-L1 <1% population was 10.9 vs. 9.3 months (HR 0.84) for nivolumab and chemotherapy, respectively. In the PD-L1 ≥ 1% population, median OS was 10.9 vs. 8.1 months (HR 0.69) for nivolumab and chemotherapy, respectively, with no significant interaction between PD-L1 status and treatment. However, the magnitude of benefit does appear higher in the PD-L1 ≥ 1% population compared to the PD-L1 negative group.

In the JAVELIN-300 Gastric Cancer study of avelumab, PD-L1 IHC was performed using the 73-10 pharmDx assay (23). PD-L1 status was defined as positive or negative based on the expression of at least ≥1% on tumor cells only (approximately equivalent to TPS ≥1%). In total, 23% was PD-L1 positive. There was no difference in OS between the PD-L1 positive and negative subgroups (4.0 vs. 4.6 months) or PFS (1.4 vs. 1.4 months), and consistently poorer than chemotherapy (PFS 2.7 months). In the JAVELIN-100 Gastric Cancer study, samples were tested for PD-L1 status using the 73-10 pharmDx assay and the 22C3 assay (44). With the 73-10 assay (≥1% on tumor cells only) 12% of patients were defined as PD-L1 positive, and with the 22C3 assay (CPS ≥1), 64% were defined as PD-L1 positive. Using the 73-10 assay, the PD-L1 positive population did not have a survival benefit for avelumab vs chemotherapy (16 vs. 18 months, HR 1.13). However, using the 22C3 assay, the CPS ≥1 population had a survival benefit for avelumab vs chemotherapy (15 vs. 12 months, HR 0.72).

From these trial results (Table 4), PD-L1 IHC measured using the 22C3 assay to calculate CPS score appears to have the best sensitivity to predict for benefit from ICI. GEC tumors with higher CPS scores tend to have higher responses and survival when treated with ICI. However, the relationship between CPS score and benefit is not linear. Rather, there appears to be a cut-off level, above which benefit occurs. This cut-off is suggested at CPS ≥1 for third-line and CPS ≥10 in first-line. Of note however, are the occurrences of responses (including CR) and prolonged survival of patients with CPS 0 (albiet at lower rates compared to higher CPS scores). This suggests that PD-L1 IHC as a stand-alone biomarker to predict for ICI benefit may be insufficient.

**Table 4:** Major GEC ICI trials.

**Name of trial**	**Tumor subtype**	**Line of Rx**	**PD-L1 IHC selection**	**PD-L1 IHC stratification**	**References**
ATTRACION-2	GC	≥3	No	No	(35)
KEYNOTE-059 Cohort 1	GC	≥3	No	NA (non randomized)	(36)
JAVELIN-300	GC	≥3	No	No	(23)
KEYNOTE-061	GC	2	No (first 83%) CPS ≥ 1 (last 17%)	Yes (CPS ≥ 1)	(25)
KEYNOTE-062	GC	1	CPS ≥ 1	No	(28)
JAVELIN-100	GC	1	No	No	(28)
KEYNOTE-181	EC	2	No	No	(15)
ATTRACTION-3	ESCC	2	No	No	(41)
KEYNOTE-180	EC	≥3	No	NA (non randomized)	(39)

### Microsatellite Instability, Mismatch Repair Deficiency and Tumor Mutational Burden

The adaptive immune system has the ability to recognize somatic mutations that occur in tumors. It is also well-established that tumor types with high levels of somatic mutations such as melanoma and lung cancer are among the most responsive to ICI (52). Mismatch repair protein (MMR) deficiency occurs in several tumor types including gastrointestinal colorectal, gastric, pancreaticobiliary, small intestine, endometrial, prostate, and ovarian cancer (53). MMR deficiency occurs through mutations in genes that recognize and correct errors in mismatched nucleotides (*MLH1, MSH2, MSH6*, and *PMS2*) or through methylation-induced gene silencing of the promoter of *MLH1*. Germline mutations in MMR proteins are associated with Lynch syndrome, although a majority of MMR deficient tumors are sporadic and occur through *MLH1* promoter methylation. The inability of the MMR proteins to function normally leads to an accumulation of errors in DNA microsatellite regions, resulting in microsatellite instability (MSI). MSI high (MSI-H) tumors tend to have several-fold higher levels of somatic mutations, and express larger numbers of predicted neoantigens (54). MSI-H tumors have demonstrated high response rates to anti-PD-1 therapy (pembrolizumab and nivolumab), with ORR > 50% (54, 55). Based on these findings, the FDA granted its first tissue agnostic approval for pembrolizumab for treatment of MSI-H tumors across any unresectable/metastatic solid tumor that had progressed following prior treatment (56). MMR deficiency can be detected either through demonstrating the loss of MMR proteins on IHC (deficient MMR, dMMR) or measuring microsatellite instability by performing PCR on prespecified microsatellite markers (MSI-H) or by enumerating known MSI loci using targeted deep next generation sequencing (MSI-NGS) (57).

In GC, MMR deficiency occurs in ~8% of early GC (stage I to stage III) and 4% in metastatic GC (54, 58). MMR deficiency occurs rarely in EC (<1%), and most EC classified as MSI-H are EAC within the GEJ areas (14). In the original basket studies of MMR deficient tumors with pembrolizumab, only a few GC cases were included. In KEYNOTE-059, 7 patients (4%) were MSI-H, and 4 had an objective response (57%) (51). In a phase II study of advanced GC conducted in South Korea, 61 patients with advanced GC that had progressed on at least one prior line of treatment were treated with single agent pembrolizumab and tumor tissues from the patients obtained prior to ICI treatment were subjected to integrated molecular analysis (59). In this study, 7 patients were MSI-H (11%) of which 6 demonstrated deep and durable responses (85%). In KEYNOTE-061, 27 patients were MSI-H, and 15 of these were randomized to the pembrolizumab arm (25). Median OS was not reached (and significantly higher) for those MSI-H patients treated with pembrolizumab (vs 8 months for paclitaxel). Similarly, ORR was higher at 47% (vs 17% for paclitaxel). In the KEYNOTE-062 study, 50 patients were found to be MSI-H, and in these, the median OS was not reached in the pembrolizumab arm and the pembrolizumab + chemotherapy arm (60). Median PFS was 11.2 months in the pembrolizumab arm and not reached in the pembrolizumab + chemotherapy arm. ORR was also significantly higher (57% for pembrolizumab and 65% for pembrolizumab + chemotherapy).

Methods have been developed to quantify the number of somatic mutations and reported as tumor mutational burden (TMB). MMR deficiency is only one of the several causes of high TMB. Mutations in several other genes such a *POLE* and *POLD1* have also been associated with high TMB (61). High TMB has also been associated with smoking-related cancers such as lung cancer and head and neck cancers, and UV-associated cancers such as melanoma (62). TMB has been traditionally measured using whole exome sequencing (WES), but performing WES in routine clinical practice is difficult. More recently, TMB has been determined reliably using contemporary next-generation sequencing (NGS) panels (63). Results of TMB are often reported as mutations/megabase (mut/Mb) (64). Various methods have been developed to report NGS panel based TMB. However, as the different NGS platforms have variations in sequencing technique and bioinformatic pipelines, there is a lack of harmonization in the quantification of TMB. Currently, there is debate and controversy on the predictive cut-off level to define high TMB across platforms (65). In lung, bladder and head and neck cancers, >200 somatic mutations detected by WES predicts consistently for response to ICI. However, the appropriate thresholds for other tumor types have not been established (66). There are clinical trials on-going assessing the role of high TMB in predicting for ICI benefit (CheckMate 848, NCT03668119 and TELMA, NCT03836066).

In GC, ~8% of tumors have high TMB, defined as >17 mut/MB in this study (53). However, GEJ tumors were found to only have a 3% prevalence of high TMB. Of the 8% of GCs that were high TMB, a majority of them were driven by MSI-H. Amongst microsatellite stable (MSS) tumors, only 1.7% of GC was found to have high TMB. In comparison, 3.5% of MSS ESCC had high TMB. In the phase II South Korean study of pembrolizumab, high TMB was defined as >400 non-synonymous single nucleotide variants (SNVs) in WES (59). Of the 61 tumors in total, 8 had high TMB (13%), of which 6 were MSI-H, one MSS and one EBV. ORR in this cohort of high TMB was 89%. In the moderate TMB group (100–400 SNVs), ORR was 20%, while in the low TMB group ORR was only 7%. In a phase I study of toripalimib, an anti-PD-1 antibody, metastatic GC patients with high TMB (> 20 mut/Mb) (20%) had a better response compared to low TMB (33 vs. 7%) and survival (15 vs. 4 months, HR 0.48) (67, 68).

**Figure 2:**
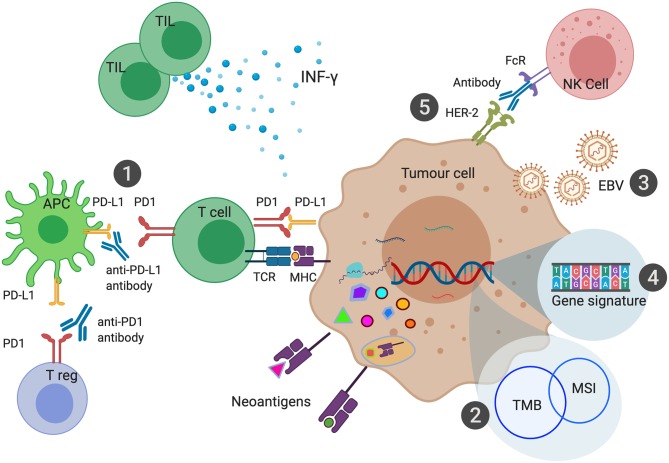
Biomarkers of Immune Checkpoint Inhibition in Gastroesophageal Cancers. PD-L1 expression can be measured in the form of Combined Positive Score (CPS) has been shown to predict response to anti-PD1 or anti-PD-L1 therapies (1). Microsatellite Instability (MSI) leads to a large number of somatic mutations and production of neoantigens. MSI is one of the most common causes of high Tumor Mutational Burden (TMB) (2). EBV associated Gastric Cancer (EBVaGC) has been postulated to be sensitive to ICI due to high intra-tumoral immune infiltration and expression of PD-L1 and PD-L2 (3). Various gene signatures have been developed to identify genes that can predict response to ICI (4). Combination of HER-2 and anti-PD1 therapy enhance antibody-dependent cellular cytotoxicity leading to improved outcomes with ICI (5).

From these studies, it is observed that there is only marginal overlap between MSI-H, TMB and PD-L1 expression. There are MSI-H tumors that have low TMB, and there are high TMB tumors which have low PD-L1 expression. Responses to ICI also do not fully correlate with any of these biomarkers, with responses occurring in CPS 0 populations and lack of response seen in patients with MSI-H and high TMB. Recent studies in MSI-H tumors have shown that the extent of response to ICI is associated with the specific accumulation of insertion-deletion mutations (69). Studies to better understand these biomarkers along with the development of other novel biomarkers are currently being pursued (Figure 2).

### Epstein-Barr Virus Positive Tumors and Sensitivity to ICI

The TCGA classification of GC identifies EBV associated gastric cancers (EBVaGC) as a unique subtype (13). EBVaGC are characterized by high intra-tumoral immune infiltration, and high transcriptomic expression of PD-L1 and PD-L2. It was postulated that EBVaGC are likely to be sensitive to ICI. The prevalence of EBVaGC in the TCGA was reported at 9%, however this study comprised predominantly of non-metastatic, resectable GC. EBVaGC tend to have a good prognosis, with low rates of nodal metastases and recurrence. The prevalence of EBVaGC in metastatic GC is likely lower than 9%, and few reports of ICI treatment of EBVaGC exist. Nevertheless, in the phase II South Korean study of pembrolizumab, six EBVaGC were included and all of six achieved a partial response (PR) to treatment, with a median duration of response of 8.5 months (59). In the phase I study of toripalimab, four EBVaGC were included and only one attained a PR. Of interest, of the four, only one was PD-L1 positive (the responder), while the other three were PD-L1 negative (67). Up to a third of EBVaGC are known to have low expression of PD-L1 and immune infiltration (70). However, given the biological rationale and clinical responses to ICI, it is reasonable to consider treating metastatic EBVaGC with single agent ICI.

### Immune Gene Signatures

Study of the interaction between the tumor and immune microenvironment has shown distinct classes of the type, location and density of immune cells within the tumor, with correlation to prognosis (71). The analysis of various immune related factors within the tumor, such as density, nature, distribution, and function is described as the “immune contexture” (72). Recently, four classes of tumors have been proposed based on the immune contexture (73). “Hot immune tumors” have high levels of cytotoxic T-cell infiltration, activation of immune checkpoints and impaired T-cell function. “Altered-immunosuppressed tumors” have some cytotoxic T-cell infiltration, but at low levels, presence of immunosuppressive Tregs and myeloid derived suppressor cells, and other inhibitory mediators such as TGFβ, IL-10, and VEGF. “Altered-excluded tumors” have no T-cell infiltration at the tumor bed, but presence of T-cells at the tumor invasive margins, epigenetic modifications within the tumor microenvironment and aberrant tumor stroma and vasculature. “Cold immune tumors” have absence of T-cells within the tumor and at the invasive margins, poor T-cell priming and resistance to T-cell mediated tumor kill. The presence of T-cells along with other factors such as interferon-γ (IFNγ), perforin and granzymes have been associated with immune functional orientation (74). The expression of various immune related genes has been developed into inflammation and immune gene signatures, which have been shown to have prognostic benefit (75–77). The predictive value of these signatures to response to ICI has been studied in the context of lung cancer and melanoma, although no signatures have been developed bespoke to GEC (78). Two of the more evolved signatures include the IFNγ-related inflammatory signature (79) and an inflammatory gene signature (80). In KEYNOTE-059, the IFNγ signature score was higher in responders compared to non-responders (51). CheckMate-032 was a phase I/II study of nivolumab with or without ipilimumab in patients with metastatic GC. In a biomarker analysis of CheckMate-032, various immune gene signatures were analyzed to predict for response to ICI (81). Of all the immune signatures tested, a 4 gene panel (*PD-L1, CD8A, LAG3, STAT1*) appeared to predict best for response in this small cohort of 40 patients. KEYNOTE-028 was a phase IB trial of pembrolizumab in patients with 20 different tumor types. In the esophageal cohort, 23 patients were enrolled, and a 6 gene IFNγ signature was tested, and showed a trend toward predicting for response to ICI (34). The role of immune gene signatures in predicting benefit from ICI in GEC continues to evolve and has not been incorporated into clinical practice.

### HER2

Overexpression of HER2 in metastatic GC occurs in ~20% of tumors and the first-line management of this subtype of GC is trastuzumab in combination with 5-FU and platinum chemotherapy (12). A group of investigators hypothesized that the combination of PD-1 and HER2 inhibition would lead to activation of T-cells and ameliorate antibody-dependent cellular cytotoxicity, leading to increase in response. Based on this hypothesis, a phase II trial of induction pembrolizumab and trastuzumab followed by 5FU/platinum/trastuzumab and pembrolizumab was conducted in patients with untreated metastatic HER2 positive GC (82). In total 35 patients were enrolled in the study, and 87% of patients had an objective response to treatment. Median PFS was 11.4 months and median OS was not reached at the time of reporting of the study. Based on these impressive results, a randomized phase III study of chemotherapy/trastuzumab +/– pembrolizumab (KEYNOTE-811) has commenced enrollment (83).

### Epigenetic Biomarkers

Epigenetic alterations have been investigated as predictive biomarkers of therapy in GEC. DNA methylation signatures have been developed to predict for chemotherapy benefit in GEC (84). In lung cancer, a DNA methylation signature was developed to predict for ICI response and validated in a separate cohort. Methylation of *FOXP1* was found to be predictive of ICI response (85). In GC, somatic epigenetic promoter alterations have been described to be involved in tumor immune editing. By altering the transcription start sites of high-affinity major histocompatibility complex class I binding GC peptides, loss of the immunogenic N-terminal peptide leads to immune evasion (86). By quantifying the utilization of the alternate promoters to generate modified isoforms, it was hypothesized that alternate promoter utilization burden could predict for response to ICI. In two separate cohorts of ICI treated GC (including the phase II South Korean study), the alternate promoter utilization burden predicted for tumors that were resistant to immunotherapy (87). These findings suggest that alternate promoter utilization burden may be a negative predictive biomarker for ICI.

## Future Direction

The landscape of immunotherapy is rapidly evolving, with the highest number of clinical trials and studies ever being conducted in this field. Multiple combinations of ICI with chemotherapy, targeted therapy and novel agents including second generation ICI continue to be tested. In the targeted therapy era, identification of driver genomic mutations and amplifications followed by blocking these drivers with drugs led to the development of relatively easy-to-test and implement biomarkers (88). However, in the immunotherapy era, there is unlikely to be a single biomarker to identify most patients that benefit from ICI. The interaction of the immune system with the tumor microenvironment, microbiome and epigenome is currently being studied at great depth and insights in these areas are likely to reveal a more complex, but deeper understanding of ICI mechanisms of response and resistance (89). It is also likely that biomarkers developed for PD-1 axis agents may not be applicable to other novel checkpoint inhibitors.

The deep and durable responses that occur with ICIs are unique to this class of drugs. Advanced tumors almost invariably develop resistance to chemotherapy and targeted therapy agents, thus rendering the intent of treatment as palliative when these are administered in the metastatic setting. However, there are now strong data emerging from patients with advanced solid cancers treated with ICI that have achieved 5-year survival, the bench mark for curative intent cancer treatment modalities (90). However, these dramatic responses occur only in a minority of patients with solid tumors (91). The negative trials that have played out in GEC are a strong reminder to the oncology community of this fact. Most of the biomarkers that have been developed for immunotherapy in GEC aim to identify tumors that are more likely to benefit from treatment. However, the remote promise of durable response has led to both patients and clinicians demanding for treatment with ICI. Perhaps, in this treatment landscape, the development of a negative predictive biomarker would be beneficial. One of the best examples of a negative predictive biomarker is the use of *RAS* mutations to predict for lack of benefit of anti-EGFR therapy in colorectal cancer (92). Similarly, for immunotherapy, it would be beneficial if immunotherapy-resistant tumors could be identified and excluded from ICI and considered for alternative therapeutic options. Given the lack of a single unified predictive biomarker for immunotherapy currently, the development of a composite biomarker could be considered, including positive predictive biomarkers such as PD-L1, MSI, TMB, HER2, and EBV status, as well as incorporating novel negative predictive biomarkers. Due to tissue considerations for processing various components of the composite biomarker (which may include IHC, genomic sequencing/profiling and fluorescent *in-situ* hybridization), industry and academic partners will need to work closely to evaluate the predictive robustness of such a biomarker in prospective clinical trials.

Immunotherapy will continue to sculpt the landscape of GEC in the coming years, and with further advances in precision oncology through the development of robust predictive biomarkers, optimal, and bespoke patient treatment strategies will emerge.

## Author Contributions

RS, JY, and SP: Drafting of the manuscript. ES and PT: Critical revision of the manuscript for important intellectual content. RS, ES, JY, SP, and PT: Final approval of manuscript.

## Conflict of Interest

RS: Advisory board: BMS, Merck, Eisai, Bayer, Taiho; honoraria for talks: MSD, Eli Lilly, BMS, Roche, Taiho; Travel funding: Roche, Astra Zeneca, Taiho, Eisai; Research funding: Paxman Coolers, MSD. ES reports personal fees from Astellas, Bristol-Myers Squibb, Celgene, Five Prime Therapeutics, Gritstone Oncology and Servier. PT and RS have submitted work discussed in this manuscript on epigenetic biomarkers as a Technology Disclosure to the institutional Technology Transfer Office, for potential intellectual property protection. The remaining authors declare that the research was conducted in the absence of any commercial or financial relationships that could be construed as a potential conflict of interest.
